# A *Solanum lycopersicum *×* Solanum pimpinellifolium* Linkage Map of Tomato Displaying Genomic Locations of R-Genes, RGAs, and Candidate Resistance/Defense-Response ESTs

**DOI:** 10.1155/2008/926090

**Published:** 2009-02-11

**Authors:** Arun Sharma, Liping Zhang, David Niño-Liu, Hamid Ashrafi, Majid R. Foolad

**Affiliations:** ^1^Institute for Plant Genomics and Biotechnology, Texas A&M University, College Station, TX 77843, USA; ^2^Nephrology Division, Department of Medicine, Baylor College of Medicine, Houston, TX 77030, USA; ^3^Monsanto Canada Inc., 3-75 Scurfield Boulevard Winnipeg, Manitoba, Canada R3Y 1P6; ^4^Department of Plant Sciences, University of California-Davis, One Shields Avenue, Davis, CA 95616, USA; ^5^Department of Horticulture, The Intercollege Graduate Degree Program in Genetics, The Pennsylvania State University, University Park, PA 16802, USA

## Abstract

We have identified an accession (LA2093) within the tomato wild species Solanum pimpinellifolium with many desirable characteristics, including biotic and abiotic stress tolerance and good fruit quality. To utilize the full genetic potential of LA2093 in tomato breeding, we have developed a linkage map based on an F_2_ population of a cross between LA2093 and a tomato breeding line, using 115 RFLP, 94 EST, and 41 RGA markers. The map spanned 1002.4 cM of the 12 tomato chromosomes with an average marker distance of 4.0 cM. The length of the map and linear order of the markers were in good agreement with the published maps of tomato. The ESTs were chosen based on their sequence similarities with known resistance or defense-response genes, signal-transduction factors, transcriptional regulators, and genes encoding pathogenesis-related proteins. Locations of several ESTs and RGAs coincided with locations of several known tomato resistance genes and quantitative resistance loci (QRLs), suggesting that candidate-gene approach may be effective in identifying and mapping new R genes. This map will be useful for marker-assisted exploitation of desirable traits in LA2093 and other *S. pimpinellifolium* accessions, and possibly for utilization of genetic variation within *S. lycopersicum.*

## 1. INTRODUCTION

The tomato *Solanum* species (*Solanum* subsection *Lycopersicon*) include the cultivated tomato, *S. lycopersicum* L. (formerly *Lycopersicon esculentum* Miller), and more than 10 related wild species (http://www.sgn.cornell.edu/about/solanum_nomenclature.pl). It is estimated that *S. lycopersicum* accounts for only about 5% of the total genetic variability in the tomato gene pool [1]. Conversely, the tomato wild species bear a wealth of genetic variability for many agriculturally and biologically important characteristics. During the past several decades, tomato wild species have been utilized extensively in traditional breeding programs, however, mainly for improvement of simply inherited traits such as vertical disease resistance. Genetic variation in the wild species for complex traits such as tolerance to environmental stresses, quantitative disease resistance, and fruit yield and quality has remained largely unexploited [2]. This is mainly due to the inadequacy of traditional breeding protocols to identify, select, and successfully transfer genes controlling such complex traits. The identification of genes underlying quantitative characters is often difficult, particularly if their phenotypic effects are unrecognizable from the phenotype [3]. Furthermore, transfer of desirable genes from wild species into elite breeding lines is not without inherent difficulties. Upon interspecific hybridization, a major task becomes eliminating the great bulk of undesirable exotic genes while maintaining and selecting for desirable characteristics. These limitations, however, may no longer be insurmountable with the advent of molecular biology tools such as genetic markers and maps and marker-assisted selection (MAS). Among various advantages, molecular markers and maps can facilitate determination of the number, chromosomal location, and individual and interactive effects of genes (or quantitative trait loci (QTL)) that affect complex traits. Following their identification, desirable genes or QTLs can be introgressed into the cultigen and undesirable characteristics eliminated by foreground and background MAS.

During the past few decades, several molecular linkage maps of tomato have been developed mainly based on interspecific crosses between the cultivated and related wild species of tomato (for a complete list see Foolad 2007). The first molecular linkage map of tomato was published in 1986, which included 18 isozyme and 94 DNA markers [4]. The high-density linkage map of tomato, which originally was developed based on an F_2_ population of a *S. lycopersicum* × *S. pennellii* cross and 1030 molecular markers [5], currently comprises more than 2000 markers with intermarker spacing of ≤1 cM (http://www.sgn.cornell.edu/cview/map.pl?map_id=9). The high level of molecular marker polymorphism between *S. lycopersicum* and *S. pennellii* facilitated the development of this high-density map. With this genetic map, it is likely that any gene of interest would be within one to a few centiMorgan (cM). However, many important agricultural traits are not segregating in this population and many of the markers in this map are not polymorphic in other populations of tomato, in particular those derived from intraspecific crosses within the cultigen or between the cultigen and closely related wild species such as *S. pimpinellifolium* L. (formerly *L. pimpinellifolium* (L.) Miller) and *S. cheesmaniae* (L. Riley) Fosberg (formerly *L. cheesmaniae* L. Riley). For example, it has been determined that only ~30% of the RFLP markers in the high-density map detect polymorphism in *S. lycopersicum* × *S. pimpinellifolium* populations following digestion of genomic DNAs with many restriction enzymes [6, 7]. In a more recent study, only less than 15% of the RFLP markers from the high-density map detected polymorphism between a Mexican accession of *S. pimpinellifolium* and a *S. lycopersicum* breeding line (MR Foolad et al., unpubl.). Such low levels of marker polymorphism necessitated the development of several species-specific molecular maps, as listed elsewhere [2]. Among the different wild species of tomato, however, genetic maps developed based on crosses between the cultivated tomato and *S. pimpinellifolium* would be more useful for practical purposes, as described below.

*S. pimpinellifolium* is the only red-fruited wild species of tomato and the only species from which natural introgression into the cultivated tomato has been detected [8]. In addition, during the past several decades, extensive genetic introgressions from this species into the cultivated tomato have been made through plant breeding [8–10]. Accessions within *S. pimpinellifolium* are highly self-compatible and bidirectionally cross-compatible with the cultivated tomato. Because of the close phylogenetic relationships between the two species, there is little or no difficulty in initial crosses or in subsequent generations of prebreeding and breeding activities. Furthermore, *S. pimpinellifolium* harbors numerous desirable horticultural and agronomic characteristics, including disease resistance [11–13], abiotic stress tolerance [14, 15], and good fruit quality [2, 16], and much fewer undesirable traits than most other wild species of tomato. However, to utilize the full genetic potential of this species, it is necessary to detect molecular polymorphisms between this species and the cultivated tomato. Detection or development of polymorphic markers, in particular functional markers (see below), and construction of new molecular linkage maps based on desirable *S. lycopersicum* × *S. pimpinellifolium* crosses are a step toward genetic exploitation of this species. Furthermore, because of extensive introgressions from *S. pimpinellifolium* into modern cultivars of tomato, such markers and maps will also be useful when exploiting the available genetic variation within the cultigen.

Most of the previous genetic linkage maps of tomato were constructed based on random genetic markers such as RFLPs, RAPDs, AFLPs, and SSRs. Recently, however, DNA sequences based on expressed sequence tags (ESTs) and resistance gene analogs (RGAs) have become available, which can be used to develop genetic markers and maps or used as candidates to identify functional genes. Development of markers and maps based on informative sequences will be useful for identification and potentially cloning of genes and QTLs of agricultural and biological significance. ESTs are generally derived from cDNA clones and may have applications in gene sighting, genome mapping, and identification of coding regions in genomic sequences. While ESTs can serve the same purposes as random DNA markers, they provide the additional feature of pointing directly to expressed genes and thus can expedite gene discovery and comparative genomics. The growing EST databases in different plant species, including tomato, have provided valuable resources for development of EST-based markers. The association of EST markers with phenotypes can lead to a better understanding of biochemical pathways and mechanisms affecting important traits. Identification and characterization of RGAs has also been proposed as a candidate-gene approach to identify genes potentially related to disease resistance [17–21]. Although not all amplified products may correspond to functional disease resistance genes [21], RGA primers have been shown to amplify the conserved sequences of leucine-rich repeats (LRR), nucleotide-binding sites (NBS), or serine/threonine protein kinases (PtoKin), thereby targeting genes and gene families for disease resistance, defense response, or other important signal transduction processes [22]. Thus, RGAs have been considered useful not only as genetic markers but also as potential that leads to the identification of important genes. During the past decade, RGAs have been used for mapping of QTLs for many important characters, including disease resistance.

Recently, we identified several accessions of *S. pimpinellifolium* (including LA2093) with desirable horticultural characteristics such as disease resistance, abiotic stress tolerance, and good fruit quality. To facilitate genetic characterization and exploitation of LA2093, and possibly other accessions, we have developed a genetic linkage map based on an F_2_ population of a cross between LA2093 and tomato breeding line NCEBR-1 using 250 DNA markers, including RFLPs, ESTs, and RGAs. Previously, two molecular linkage maps of tomato based on different crosses between *S. lycopersicum* (denoted as L) and *S. pimpinellifolium* (denoted as PM) were reported by Grandillo and Tanksley [6] (referred to as L × PM1 map) and Chen and Foolad [7] (referred to as L × PM2 map). The map presented here (referred to as L × PM3) is different but complementary to the previous two L × PM maps, as it contains a large number of ESTs and RGAs along with some new RFLP anchor markers that can facilitate molecular investigation and exploitation of this and other accessions of *S. pimpinellifolium*. We have compared the L × PM3 map with other molecular linkage maps of tomato and discussed similarities and differences in relation to phylogenetic relationships between parents of the various mapping populations.

## 2. MATERIALS AND METHODS

### 2.1. Plant materials and mapping population

Inbred sources of NCEBR-1 (*S. lycopersicum*) and LA2093 (*S. pimpinellifolium*) were hybridized and F_1_ progeny produced. NCEBR-1 (PVP) is a horticulturally superior, multiple disease resistant, advanced tomato breeding line received from RG Gardner, University of North Carolina, Fletcher, NC, USA. A single F_1_ hybrid plant was self-fertilized to produce F_2_ seed. A total of 900 F_2_ individuals were grown under field conditions and screened for various characteristics. Among other traits, the population was segregating for growth habit (determinate versus indeterminate). Indeterminate growth habit is an undesirable characteristic with confounding effects on other characteristics such as disease resistance and fruit quality. To obtain a population suitable for QTL mapping and breeding purposes, indeterminate plants were eliminated. A total of 172 F_2_ individuals, hereafter referred to as the L × PM3 F_2_ population, were chosen and grown to maturity and used to construct the molecular linkage map.

### 2.2. RFLP analysis

Nuclear DNA was extracted from approximately 10 g of leaf tissue from each of the parental lines and F_2_ individuals using standard protocols for tomato [23, 24]. Genomic DNAs were treated with RNase and digested with eight restriction enzymes, including *Dra*I, *Eco*RI, *Eco*RV, *Hae*III, *Hind*III, *Rsa*I, *Sca*1, and *Xba*1 following manufacturers' instructions, and parental polymorphism survey blots were prepared. To identify sufficient number of polymorphic anchor RFLP markers to develop a framework linkage map, parental survey blots were probed with a total of 340 random tomato genomic (TG) or cDNA (CD or CT) clones, originally chosen from the high-density molecular linkage map of tomato [25]. Agarose gel electrophoresis, Southern blotting, hybridizations, and autoradiography were conducted as described elsewhere [26]. Probes were labeled with [^32^P]dCTP by primer extension [27]. Following identification of polymorphic RFLP markers (see Section 3 for rates of polymorphism), genomic DNAs of the 172 F_2_ individuals were digested with the 8 restriction enzymes and multiple sets of Southern blots were prepared. Blots were hybridized with clones detecting polymorphism and a total of 115 RFLP markers were scored in the F_2_ population.

### 2.3. EST analysis

A set of unique ESTs was selected from the tomato gene index sources maintained by The Institute for Genomic Research (TIGR; http://www.tigr.org/) (now at the Computational Biology and Functional Genomics Laboratory at Harvard University; http://compbio.dfci.harvard.edu/tgi/cgi-bin/tgi/est_report.pl). Each EST represents a valid (partial or complete) copy of a transcribed functional allele. We selected 140 ESTs from a diverse array of candidate genes and gene families, many of which are known or assumed to play roles in disease-resistance or defense-response mechanisms. Among them we included ESTs with homology to resistance (*R*) genes, signal transduction genes, transcriptional regulator factors, and genes encoding pathogenesis-related proteins. We used this targeted strategy to obtain a set of potentially functional markers for marker-assisted selection in our tomato-breeding program. The 140 EST clones, purchased from the Clemson University Genomics Institute (http://www.genome.clemson.edu/), Clemson, SC, USA, were used as RFLP probes to identify polymorphism between the two parents. Among them, 96 provided polymorphic alleles (Table 1). The polymorphic ESTs were used as RFLP probes to genotype the F_2_ individuals, examine their segregation, and map onto the tomato chromosomes.

### 2.4. RGA analysis

#### 2.4.1. Selection of primers

Ten pairs of oligonucleotide primers, previously designed based on conserved LRR, NBS, and PtoKin motifs of several resistance genes, were used (Table 2; [28]). Some primers were chosen to be degenerate at the redundant third position (3′ end) in the codons to cover a range of possible sequences encoding the motifs, and thus to increase the efficiency of PCR amplification [19, 29]. Only one pair of primers was used for each PCR amplification.

#### 2.4.2. PCR amplification

PCR conditions for amplification of RGAs were described elsewhere [11]. Briefly, each amplification was performed in a 25-*μ*L volume consisting of 300 *μ*M each of dATP, dCTP, dGTP, and dTTP, 5 mM of MgCl_2_, 1 unit of *Taq* DNA polymerase, 2.5 *μ*L of 10X buffer (PCR Core system I; Promega, Madison, Wis, USA), 2 *μ*L of each primer, and 40 ng of genomic DNA that was used as template. For control reactions, the template was substituted with sterile, nuclease-free ddH_2_O. All PCR mixtures were overlaid with mineral oil and carried out in a Perkin Elmer DNA Thermal Cycler 480 (Perkin Elmer, Foster City, Calif, USA), programmed for 4 minutes at 94°C for an initial denaturation, and 36 cycles of 1 minute at 94°C (DNA denaturation), 1 minute at 50°C (primer annealing), and 1.5 minutes at 72°C (primer extension), followed by a final 7-minute extension at 72°C.

#### 2.4.3. Gel electrophoresis and silver staining

Denaturing polyacrylamide gel electrophoresis (PAGE) was used to separate and detect individual RGA bands [30]. Briefly, a denaturing gel (7 M Urea—6% polyacrylamide) was prepared in a sequencing gel apparatus (420 × 330 × 0.4 mm; Fisher Biotech, Springfield, NJ, USA) using Bind- and Repel-Silane (Promega). After polymerization, the gel was prerun in 1X Tris-borate-EDTA (TBE) buffer for 30 minutes at 40 W (~1400 V) to reach a gel temperature of 50°C. Twelve *μ*L of loading buffer (10 M Urea—0.08% xylene cyanole) were added to each 25 *μ*L amplified DNA sample and the mixture was denatured at 95°C for 5 minutes and immediately put on ice. After cleaning the gel-loading surface, a 0.4 mm-thick shark comb (Fisher Biotech, Springfield, NJ, USA) was inserted into the gel. Subsequently, 7 *μ*L of each PCR-amplified sample were loaded. Each gel accommodated 60 DNA samples and three DNA size markers (1 Kb, 100 bp, 50 bp). The gel was run at 35 W (~1350 V) for 3.5–4 hours. After electrophoresis, the gel, fixed to the Bind-Silane surface of one glass plate, was silver stained following the manufacturer's protocol (Promega). The gel was air dried at room temperature overnight and stored in dark for future scoring and scanning. All amplifications and gel electrophoresis procedures were repeated at least once.

#### 2.4.4. Identification of informative RGA markers

Following gel electrophoresis and staining, polymorphic and monomorphic bands were observed. Polymorphic bands were directly scored as dominant markers and used for genetic mapping. To determine whether monomorphic bands could detect polymorphism if used as RFLP probes, they were excised from the gel (as described in [28, 32]), purified with the QIAgene quick Gel Extraction Kit (QIAGEN, Valencia, Calif, USA), labeled with ^32^P-dCTP, and used to hybridize the parental survey blots. Probes which detected polymorphism between the two parents were then used to hybridize Southern blots of the F_2_ population, and scored as either dominant or codominant markers. Overall, a total of 43 RGA markers were successfully scored and mapped onto the 12 tomato chromosomes.

#### 2.4.5. Size determination of RGA fragments

 PAGE polymorphic and monomorphic fragments were excised from the dried polyacrylamide gel and reamplified, by using a needle scratching and PCR reamplification method [32]. The reamplified products and DNA size markers (1 Kb, 100 bp, and 50 bp) were run on a 1.0% agarose gel, stained with ethidium bromide, and photographed to determine the size.

### 2.5. Statistical and mapping analyses

Segregation of the 250 DNA markers (115 RFLPs, 94 ESTs, and 41 RGAs) in the F_2_ population was tested for deviation from the expected Mendelian genotypic ratios of 1 : 2 : 1 (for codominant) or 1 : 1 (for dominant markers) using chi-square (*χ* ^2^) goodness-of-fit analysis. Multipoint linkage analysis of the genetic markers in the F_2_ population was performed using the MapMaker program v. 3.0 [33] and a genetic linkage map was constructed. Briefly, the group command was used to assign markers into linkage groups using a minimum LOD score of 3.0 and a maximum recombination fraction of 0.20. Three-point linkage analysis was performed to determine the maximum likelihood recombination fraction and the associated LOD score for each combination of loci. The “order” and “compare” commands were used to find the best order of loci within each group, followed by using the “ripple” command to verify the order. Markers were included within the framework map only if the LOD value for the ripple was greater than 3.0. Once the linear order of markers along each chromosome was determined, recombination frequencies between markers were estimated with multipoint linkage analyses. The Kosambi mapping function [34] was used to convert recombination frequencies to map distances in cM. The distribution of percentage of the *S*. *lycopersicum* genome (L) in the F_2_ population was estimated using the computer program Qgene [35].

## 3. RESULTS AND DISCUSSION

### 3.1. RFLP polymorphism between S. lycopersicum and S. pimpinellifolium

RFLP clones were chosen from two sources, a previously published *S. lycopersicum* (NC84173) × *S. pimpinellifolium* (LA722) linkage map (L × PM2) [7] and the high-density *S. lycopersicum* (VF36 *-Tm2^a^*) × *S. pennellii* (LA716) linkage map of tomato (L × P) [25]. Of the 152 RFLP clones chosen from the L × PM2 map, 82 (54%) were polymorphic between the two parents (NCEBR-1 and LA2093) in the present study. Of the 120 clones that were chosen based on the high-density L × P map, 40 (30%) were polymorphic between NCEBR-1 and LA2093. The latter level of polymorphism was similar to those previously reported by Grandillo and Tanksley [6] and Chen and Foolad [7] for different *S. lycopersicum* × *S. pimpinellifolium* crosses. A lower level of DNA polymorphism between *S. lycopersicum* and *S. pimpinellifolium* compared to that between *S. lycopersicum* and *S. pennellii* was expected as *S. pimpinellifolium* is phylogenetically much closer to the cultivated tomato [1, 36, 37]. The high-density map of tomato was constructed based on A *S. lycopersicum* × *S. pennellii* cross mainly because of the presence of high level of marker polymorphism between the two species. However, identification of polymorphic markers and development of maps based *S. lycopersicum* × *S. pimpinellifolium* crosses are essential to facilitate marker-assisted exploitation of genetic variation present in *S. pimpinellifolium*. Such information may also be useful for exploitation of intraspecific variation within *S. lycopersicum*. This is because of frequent introgressions from *S. pimpinellifolium* into the cultivated tomato,which have occurred both naturally and deliberately via plant breeding [8]. In the present study, a total of 117 polymorphic RFLP clones were used to construct the backbone linkage map.

### 3.2. EST polymorphism between S. lycopersicum and S. pimpinellifolium

From a total of 140 tomato ESTs examined, 91 (65%) were polymorphic between the two parents. Five of 91 EST clones produced more than one polymorphic band, thus resulting in the detection of a total of 96 polymorphic EST loci, including 91 codominant (~95%) and 5 dominant markers. Of the 96 EST markers, 94 were successfully scored in the F_2_ population and mapped onto the 12 tomato chromosomes using the 115 RFLP anchor markers. The number of EST markers per chromosome ranged from 4 (on chr. 12) to 12 (on chr. 10). Observation of a high level of polymorphism in EST markers between *S. lycopersicum* and *S. pimpinellifolium* was unexpected, but encouraging. This high level of polymorphism could be due to various reasons including high copy number of EST bands (compared to the often single-copy RFLP markers) and the nature of the genes or gene families from which ESTs were selected. As indicated earlier, most ESTs were chosen based on their sequence similarities with genes or proteins related to disease resistance. It is likely that chromosomal regions containing resistance gene families accumulate a great deal of variation during their evolution, thus increasing the frequency of restriction sites, which are a basis for polymorphism. Because modern breeding lines have received frequent introgressions from different tomato wild species, in particular for disease resistance, presence of such introgressions in NCEBR-1 could have contributed to the high level of observed polymorphism. Further inspections of the chromosomal locations of ESTs support this submission, as discussed below. However, the observation of high level of EST polymorphism is promising as larger number of ESTs are becoming available.

### 3.3. Marker segregation

Of the 250 marker loci scored in the L × PM3 F_2_ population, 41 (16.4%) exhibited significant deviation from the expected 1 : 2 : 1 (codominant) or 1 : 1 (dominant) segregation ratios at *P* ≤ .01. Markers with skewed segregation were located on chromosomes 1, 3, 4, 5, and 6, with those on chromosome 6 exhibiting the highest level of skewness (Table 3). Markers on chromosomes 1, 3, and 4 exhibited distortion in favor of *S. pimpinellifolium* alleles whereas those on chromosomes 5 and 6 were in favor of *S. lycopersicum*. Observation of extensive segregation distortion for markers on chromosome 6 was not unexpected and could be attributed to the selection of determinate F_2_ plants (as described in Section 2) and the presence of self-pruning (*sp*) locus on this chromosome (~3 cM from RFLP marker TG279) [6]. Skewed segregation for markers on this chromosome was previously reported in other interspecific crosses of tomato, where phenotypic selection (PS) or MAS was employed to remove indeterminate plants from mapping populations [28, 38, 39]. However, in the present study, despite skewed segregation for markers on chromosome 6, no major differences in genetic map distances were observed when they were compared with the high-density map of tomato [39] or the previous *S. lycopersicum* × *S. pimpinellifolium* maps [6, 7], where no such selections were practiced.

Skewed segregation has been reported in many interspecific crosses of tomato, with the extent of skewness being greater in wider crosses compared to crosses between closely related species, and also generally greater in F_2_ than in backcross populations [6, 40–45]. A survey of recently published results of interspecific crosses of tomato indicated that skewed segregation was 8.3% in the L × PM1 BC_1_ population [6], 9.9% in the L × PM2 BC_1_ population [7], 51% in a *S. lycopersicum* × *S. cheesmaniae* (L × CH) F_2_ population [42], 69% in a *S. lycopersicum* × *S. chmielewskii* (L × CL) BC_1_ population [46], 15% in a *S. lycopersicum* × *S. habrochaites* (L × H1) BC_1_ population [38], 62% in the L × H2 BC_1_ population [28], and 80% in a *S. lycopersicum* × *S. pennellii* (L × P) F_2_ population [47]. The L × PM populations exhibited less overall skewed segregation than the other interspecific crosses, consistent with the close phylogenetic relationship between *S. lycopersicum* and *S. pimpinellifolium*. However, the relatively high level of skewed segregation in the L × CH F_2_ population [42] and the low level of skewed segregation in the L × H1 BC_1_ populations [38] were unordinary because *S. cheesmaniae* is a closely related and *S. habrochaites* is a distantly related wild species of tomato [1, 9, 10, 48, 49]. Skewed segregation in interspecific crosses of tomato has been attributed to various causes, including self-incompatibility (SI), unilateral incongruity, and gametophytic, zygotic, and viability selection in segregating populations, as discussed elsewhere [44, 50–52].

### 3.4. Genome composition of the F_2_ population

**Figure 1:**
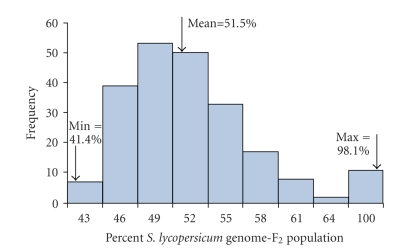
Distribution of percent *Solanum lycopersicum* genome in the F_2_ population, estimated based on 220 codominant markers.

The genomic compositions of the 172 F_2_ individuals were determined based on the 220 codominant markers using qgene program. On average, the F_2_ population was inferred to contain 51.5% of its genome from the *S. lycopersicum* parent (L alleles), which is very close to the expected 50%. The percent L genome of individual F_2_ plants ranged from 41.4% to 97.8% (Figure 1), indicating the high level of variation in the F_2_ population. This analysis clearly demonstrates the power of marker genotyping for precise determination of the genomic composition of individual plants in breeding populations. Such information can facilitate the selection of suitable plants and introgression of desirable and elimination of undesirable chromosomal segments in genetic populations derived via backcross breeding. For example, in the present population, individuals with ≥65% L genome (Figure 1) could be returned to nearly 100% L genome within 2–4 backcrosses, far more rapid than the 4–6 backcrosses routinely needed to eliminate donor genome without MAS. Alternatively, in a pedigree-type breeding program, marker analysis (if economically feasible) can facilitate inbreeding to homozygosity by selecting progeny at each generation which are homozygous over a maximal proportion of the genome.

### 3.5. Construction of the linkage map

**Figure 2:**
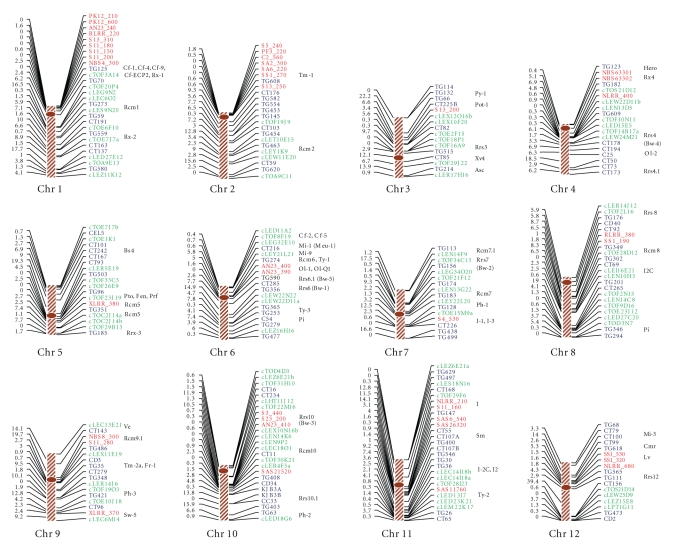
A genetic linkage map of tomato constructed based on an F_2_ population of a cross between a tomato (*S. lycopersicum*) breeding line (NCEBR-1) and an accession (LA2093) of tomato wild species *S. pimpinellifolium* and 250 RFLP, EST, and RGA markers. RFLP markers are shown in blue, ESTs in green, and RGAs in red fonts. The names of the markers are shown at the right and the map distances between them (in cM, using Kosambi function) are shown at the left of the chromosomes. The approximate chromosomal locations of disease-resistance genes (*R*-genes) and quantitative resistance loci (QRL), as inferred from other published researches, are shown in parentheses to the right of chromosomes. The descriptions of the *R*-genes and QRL are as follows: *Asc*: resistance to Alternaria stem canker (*Alternaria* alternata f. sp. *lycopersici*) [54, 55]; *Bw* (*1–5*) or *Rrs* (*3–12*): QLRs for resistance to bacterial wilt (*Ralstonia solanacearum*) [56–59]; *Cf* (*1–9, ECP2*): resistance to leaf mould (*Cladosporium fulvum*) [60–65]; *Cmr*: cucumber mosaic virus [66]; *Fen*: sensitivity to herbicide fenthion [67]; *Frl*: resistance to Fusarium crown and root rot (*Fusarium oxysporum f. sp. radicis-lycopersici*) [68]; *Hero*: resistance to potato cyst namatode (*Globodera rostochiensis*) [69]; *I* (*I,* *1, 2, 2C, 3*): resistance to different races of Fusarium wild (*Fusarium oxysporum f. sp. lycopersici*) [70–78]; *Lv*: resistance to powdery mildew (*Leveuillula taurica*) [79]; *Meu-1*: resistance to potato aphid [80–82]; *Mi* (*Mi, 1, 2, 3, 9)*: resistance to root knot nematodes (*Meloidogyne* spp.) [81, 83–89]; *Ol* (*1, 2, 3*): resistance to powdery mildew (*Oidium lycopersicum*) [90, 91]; *Ph* (*1, 2, 3*): resistance to late blight (*Phytophthora infestans*) in tomato [92–94]; *Pot-1*: resistance to potyvirus [95]; *Pto* and *Prf*: resistance to bacterial speck (*Pseudomonase syringae* pv *tomato*) [96, 97]; *Py-1*: resistance to corky root rot (*Pyrenochaeta lycopersici*) [98]; *Rcm* (*1–10*): QRL for resistance to bacterial canker (*Clavibacter michiganensis*) [99, 100]; *Rrs* (*3–12*) or *Bw* (*1–5)*: QLRs for resistance to bacterial wilt (*Ralstonia solanacearum*) [56–59]; *Rx* (*1, 2, 3, 4*): resistance to bacterial spot (*Xanthomonas campestris*) [101–103]; *Sm*: resistance to *Stemphilium* [104]; *Sw-5*: resistance to tomato-spotted wilt virus [105, 106]; *Tm-1* and *Tm-2^a^*: resistance to tobacco mosaic virus [68, 107–110]; *Ty* (*1, 2, 3*): resistance to tomato yellow leaf curl virus [111–113]; *Ve*: resistance to *Verticillium dahliae* [114, 115].

A genetic linkage map was constructed based on 115 RFLP, 94 EST, and 41 RGA loci using the F_2_ population of 172 individuals. The present map (L × PM3) spanned 1002.4 cM of tomato genome with an average marker interval length of 4.0 cM (Figure 2). The number of markers per chromosome ranged from 16 (chrs. 3 and 7) to 28 (chr. 1). Chromosome 1 had the largest linkage group (102.9 cM) followed by chromosomes 9 and 2 (96.1 and 92.6 cM, resp.), whereas chromosome 7 had the smallest one (69.8 cM), preceded by chromosomes 4 and 5 (72.2 and 70.6 cM, resp.). Only two regions, on chromosomes 3 and 12, contained marker intervals larger than 20 cM (Figure 2), and this was mainly because of the low level of polymorphism between the two parents of this mapping population for markers on these chromosomes. This map was compared with several other molecular linkage maps of tomato for marker order, recombination frequencies, and total map length, as described below.

### 3.6. Mapping of ESTs

The use of the 115 RFLP anchor markers facilitated mapping of the 94 EST loci onto the 12 tomato chromosomes. The number of ESTs per chromosome ranged from 4 (chr. 12) to 12 (chr. 10) (Figure 2). The use of ESTs as genetic markers has several advantages. First, they can be used as codominant markers for genetic mapping and QTL identification [53]. Although ESTs were used as RFLP markers, that is, through Southern hybridization, technically they can be converted to PCR-based markers adapted to high-throughput analysis. Such conversion may reduce polymorphism level, in particular between closely related individuals, though it is expected to enhance their utility as genetic markers. Second, mapping of ESTs can facilitate association of functionality with phenotype. EST markers are derived from partial or complete sequences of cDNA clones, which may provide information on gene function. Third, coding sequences, especially those of house-keeping genes, are rather conserved across species. Mapping of ESTs and comparative genomics may lead to the detection of new genes in different species.

Inspections of the distribution of ESTs on different chromosomes indicated that in some cases they were clustered, for example, ESTs on chromosomes 4, 8, 10, and 11. Further inspections indicated that chromosomal locations of some clustered or individual ESTs were colocalized with approximate locations of some major disease-resistance genes (*R*-genes) or quantitative resistance loci (QRLs), as inferred from other published researche (see Figure 2). While such colocalization suggests that these ESTs may be genetically related to resistance genes or QRL, their actual functionality relationships can only be determined by further analyses such as isolation and sequencing of full EST sequences and functional genomic studies.

Currently, there are more than 214 000 ESTs identified in tomato (http://compbio.dfci.harvard.edu/tgi/cgi-bin/tgi/gimain.pl?gudb=tomato), of which only a small percentage has been mapped onto the tomato chromosomes (http://www.sgn.cornell.edu/cgi-bin/search/direct_search.pl?search=EST). The ESTs were derived from more than 23 cDNA libraries [116, 117] and their sequences are available on Solanaceae Genome Network (SGN; http://www.sgn.cornell.edu/). All but four (cLET10E15, cLER4F5, cLEC6O2, and cLEG9N2) of the ESTs mapped in the present study were not previously mapped onto tomato chromosomes. Moreover, of the four that were previously mapped, different members of the corresponding contigs were mapped onto the same or different tomato chromosomes as in the present study. For example, cLET10E15 and cLER4F5 have overlap sequences with cLET1A5 and cLET3F16, respectively, and were mapped on the same chromosomes (http://www.sgn.cornell.edu/) as in the present study. cLEC6O2, which was mapped to chromosome 1 in the present study, was mapped to chromosome 8 and named cLPT1J10 (SGN: F_2_ population ofa cross *S. lycopersicum* LA925 × *S. pennellii* LA716). EST clone cLEG9N2, which was mapped to chromosome 1 in this study, was previously mapped under cLET20B4 but with no known chromosomal position (http://www.sgn.cornell.edu/). Also, as indicated earlier, five of the EST clones resulted in two pairs of polymorphic bands each. For two of these clones, the two polymorphic bands were mapped onto two linked loci, that is, cTOC2J14a and cTOC2J14b on chromosome 5 and cLEC14I18a and cLEC14I18b on chromosome 11. Others were mapped onto different chromosomes; for example, cLEW22D11a was mapped to chromosome 6 whereas cLEW22D11b to chromosome 4, and cTOE7J7a was mapped to chromosome 1 whereas cTOE7J7b to chromosome 5.

### 3.7. Mapping of RGAs

PCR amplification using the 10 pairs of RGA primers (Table 2) followed by denaturing PAGE resulted in the detection of a few hundred polymorphic and monomorphic bands. As described in Section 2, of the detected bands, 41 were strong and verifiable and thus were scored in the F_2_ population. The amplified fragment size of these RGA bands ranged from 150 to 760 bp. Linkage analysis indicated that the 41 RGA markers were located on the 12 tomato chromosomes, ranging in number from 1 (on chrs. 3, 5, and 7) to 9 (on chr. 1) (Figure 2). The results indicated that RGA loci could be used as genetic markers for genome mapping, consistent with previous suggestions [28, 30]. In several cases, RGA loci were clustered, similar to that observed for *R*-genes in various plant species [17, 19, 29, 60, 118–120]. For example, on each of chromosomes 1, 2, 9, 10, 11, and 12, three or more RGA loci that were amplified from the same or different primer pairs mapped to the same or nearby positions (Figure 2). This observation indicated that different primers might initiate amplification of closely linked RGA loci that might be members of the same or different gene families.

Map positions of RGA loci were compared with chromosomal positions of known tomato *R*-genes and major QRL, whose positions were inferred from the previously published maps, as displayed and described in Figure 2. Most positions were inferred based on linkage to reference markers and thus should be considered best approximations. Colocalization of RGA loci with *R*-genes and QRLs were observed on a few chromosomes, including regions on chromosomes 1, 2, 7, 8, 9, 10, 11, and 12 (Figure 2). These observations suggest the possibility of the presence of *R* or DR genes at the locations of RGAs, though this hypothesis could be confirmed only by extensive mapping and functional analysis of RGAs. Specifically, mapping of the associated RGAs in populations segregating for the colocalized *R*-genes and cloning and molecular characterization of RGAs are necessary before any functional relationship could be established.

The map positions of the RGA loci in the present map (L × PM3) were compared with those reported in a *S. lycopersicum * × * S. habrochaites* (L × H2) map [28]. There were 19 common RGA loci between the two populations and 13 (68%) of which mapped to the same locations in the two maps, suggesting consistent and reproducible positions of RGAs across populations.

### 3.8. Comparison of the map with other molecular linkage maps of tomato

The present map (L × PM3) was compared with two previously developed *S. lycopersicum* × *S. pimpinellifolium* maps, including L × PM1 [6] and L × PM2 [7] as well as the high-density *S. lycopersicum* × *S. pennellii* (L × P) map of tomato [25]. The present map is different but complementary to L × PM1 and L × PM2 maps in several ways. First, different *S. lycopersicum* and *S. pimpinellifolium* parents and pretty much different molecular markers were used in the construction of the three maps. The L × PM1 was constructed based on a cross between a processing tomato cultivar (M82-1-7) and *S. pimpinellifolium* accession LA1589 using ~120 RFLP and RAPD markers. The L × PM2 was constructed based on a cross between a fresh market tomato breeding line (NC84173) and *S. pimpinellifolium* accession LA722 using 151 RFLP markers. The current map (L × PM3) was constructed based on 250 RFLP, EST, and RGA markers using superior parental lines, as described earlier. It is expected that this map will have great utilities, including exploitation of the genetic potential of LA2093 and other *S. pimpinellifolium* accessions.

The second point of difference is that relatively a small percentage of the markers used in the present study were used in the previous two *S. lycopersicum* × *S. pimpinellifolium* linkage maps. Specifically, a new set of RFLP clones that detect polymorphism between *S. lycopersicum* and *S. pimpinellifolium* has been identified in the present study, beyond those that were identified in the construction of the previous two maps. However, an important observation is that markers that are polymorphic in one L × PM cross usually have a greater chance of being polymorphic in other L × PM crosses, compared to markers directly chosen from the high-density L × P map. Nonetheless, the observation that only 54% of the mapped RFLP clones in the L × PM2 population were polymorphic in the L × PM3 population indicates the presence of considerable DNA sequence variation among *S. pimpinellifolium* accessions. The overall results suggest that while for each *S. pimpinellifolium* accession new polymorphic markers need to be identified, the most useful sources would be those markers that have already been mapped in other *S. lycopersicum* × *S. pimpinellifolium* crosses. Third, unlike in the previous two L × PM maps, in the present map, “functional” markers such as ESTs and RGAs were used. Such markers may be more useful than random genetic markers for identification of candidate genes. The use of a large number of markers and the incorporation of functional markers in the present map extends its practical value in various genetics and breeding studies. However, the availability of three L × PM maps with rather different molecular markers should facilitate marker-assisted exploitation of these and other *S. pimpinellifolium* accessions.

When the current map was compared with L × PM1 [6], L × PM2 [7], and the high-density L × P map [25], it was determined that the linear order of the common markers were generally the same. However, there were differences in interval lengths for several adjacent markers. For example, of 13 common marker intervals between L × PM3 and L × PM2 maps, 6 intervals on chromosomes 2, 3, 5, 6, and 12 differed in length by 2-3 fold, of which 1 interval was expanded in L × PM3 map. The difference between the two maps in marker interval lengths was not unexpected given the use of different type populations (F_2_ versus BC_1_), rather small size populations (172 and 119) and different number of markers (250 versus 151), all of which could have affected the occurrence and detection of recombination in different intervals. When the L × PM3 was compared with the high-density L × P map, which was constructed based on >1 000 genetic markers and 67 F_2_ plants, genetic distances differed markedly for a large number of marker intervals. For example, for 36 common marker intervals, genetic distances differed between the two maps by at least twofold; of these, 7 intervals (23%) showed decreased and 13 (36%) showed increased recombination in the L × PM3 map. Greater differences in marker interval lengths between L × PM3 and L × P maps compared to that between L × PM3 and L × PM2 maps was not unusual considering the relatively close phylogenetic relationships between the L × PM3 and L × PM2 mapping populations.

When comparing the L × PM3 map with the high-density L × P map, the most striking differences in genetic distances were observed in centromeric regions of chromosomes 3, 4, and 9, where substantial expansions in map distances were observed in the L × PM3 map, and in two locations on chromosome 12, where substantial contractions were observed in the L × PM3 map (Table 4 and Figure 1). The decrease in recombination frequencies in the centromeric regions of tomato chromosomes was previously attributed to the centromeric suppression of recombination [5, 121, 122]. Such suppression was suggested to be more frequent in wider crosses than in intraspecific crosses and crosses between closely related species. Further inspections indicated that the differences in genetic distances between the two maps across the rest of the genome were generally interval specific and not a characteristic of individual chromosomes. For example, for chromosomes 2, 3, 6, 10, 11, and 12, the L × PM3 map exhibited expansion in some intervals and contraction in others (Table 4). As indicated earlier, such differences were due in part to the detection of chance recombination given the limited population sizes used in these studies.

Comparisons were also made across the four maps (L × PM3, L × PM2, L × PM1, and L × P) in terms of individual chromosome and total map lengths. The total length of the current map (1002 cM) was comparable with that of the L × P (1277 cM), L × PM1 (1275 cM), and the L × PM2 (1186 cM) maps. Furthermore, across the maps the length of each chromosome in the current map was comparable to the corresponding chromosome in the other maps (Table 5).

## 4. CONCLUSION

A medium-density molecular linkage map of tomato is developed based on a cross between *S. lycopersicum* and *S. pimpinellifolium*, two phylogenetically closely related species. The parents of this map are superior genotypes and are expected to be useful for tomato crop improvement. This map will provide a basis for the identification, characterization, and introgression of useful genes and QTLs present in LA2093 and other *S. pimpinellifolium* accessions. It will also facilitate studies of gene and genome organization and evolution, dissection of complex traits, and targeted gene cloning. The map includes different types of molecular markers and provides a basis for identifying and adding other markers. The genomic locations of several EST and RGA markers coincided with locations of several known tomato *R*-genes or QRL, suggesting that candidate gene approach may be an effective means of identifying and mapping new *R*-genes and defining the genetic content of specific chromosomal regions. Because of the close phylogenetic relationship between the two species and the past frequent introgression of DNA from *S. pimpinellifolium* into *S. lycopersicum*, this map is expected to be particularly useful to breeding programs that exploit intraspecific variability within the cultivated tomato. The combined information from this and the two previously published *S. lycopersicum* × *S. pimpinellifolium* maps will facilitate further identification and exploitation of genetic variation within *S. pimpinellifolium*, *S. lycopersicum* var. *cerasiforme*, and *S. lycopersicum*.

## Figures and Tables

**Table 1:** Listof ESTs mapped in the *Solanum lycopersicum × S. pimpinellifolium* F_2_ population, their putative function, chromosomal location, and copy number.

EST clone	^a^SGN-ID	^b^Putative function	Chr.	^c^Copy no.
cTOF3A14	C146883	Cytosolic Cu, Zn Superoxide dismutase, *S. lycopersicum*	1	2
cTOE7J7a	C139397	Endo-1,4-beta-glucanase, *S. lycopersicum*	1	6
cLED27E12	C19568	Cold acclimation protein WCOR413-like protein form, *O. sativa*	1	2
cTOE6F10	C139034	Lipoxygenase, *S. lycopersicum*	1	5
cLEG9N2	C45935	Subunit A of ferredoxin-thioredoxin reductase, *S. tuberosum*	1	1
cLES9N20	C79709	ASC1 (Alternaria stem canker resistance protein), *S. lycopersicum*	1	1
cLEC6O2	C11013	Polyamine oxidase, *A. thaliana*	1	1
cTOF20P4	C142906	Carotenoid cleavage dioxygenase 1-2, *S. lycopersicum*	1	5
cLEZ11K12	C98684	Snakin2 precursor, *S. lycopersicum*	1	1
cTOA9E13	C117653	Squalene synthase, *C. annuum*	1	5
cTOA9C11	C117644	Similar to WRKY transcription factor Nt-SubD48, *N. tabacum*	2	1
cLET10E15	C79822	Acidic 26kDa endochitinase precursor, *S. lycopersicum*	2	1
cTOF19J9	C142319	Phosphoribosylanthranilate isomerase, *A. thaliana*	2	1
cLEY1K9	C97179	Pathogen-inducible alpha-dioxygenase, *N. attenuata*	2	4
cLEW11E20	C89000	Resistance complex protein I2C-3, *S. lycopersicum*	2	7
cTOF16A9	C141311	Calmodulin 3 protein, *S. lycopersicum*	3	9
cLER17H16	C71298	Elicitor-inducible cytochrome P450, *N. tabacum*	3	1
cTOF18P1	C142154	Serine palmitoyltransferase, *S. tuberosum*	3	3
cLEX12O16	C92852	Ethylene response factor 5, *S. * *lycopersicum*	3	6
cTOE2F15	C137984	Catalase isozyme 1, *S. lycopersicum*	3	1
cTOF29J22	C145412	4-coumarate-coA ligase 1, *S. tuberosum*	3	2
cLEX10F20	C92172	Ethylene response factor 1, *S. lycopersicum*	3	4
cTOF14B17	C141010	Anthocyanin 5-O-glucosyltransferase, *S. sogarandinum*	4	1
cLED15E5	C16128	Shikimate kinase chloroplast precursor, *S. lycopersicum*	4	1
cLEN13D5	C66215	Chorismate synthase 1 precursor, *S. lycopersicum*	4	4
cTOS21D12	C163577	Similar to heat shock factor, *N. tabacum*	4	3
cTOF10N11	C140057	Myo-inositol-1-phosphate synthase, *S. lycopersicum*	4	4/5
cLEW24M21	C90911	TMV disease resistance protein-like protein, *Cicer arietinum*	4	2
cLEW22D11b	C90352	4-coumarate:coenzyme A ligase, *N. tabacum*	4	10
cLER5E19	C73560	Phospholipase PLDb1, *S. lycopersicum*	5	1
cTOC2J14a	C127676	Disease resistance gene homolog Mi-copy1, *S. * *lycopersicum*	5	9
cTOC2J14b	C127676	Disease resistance gene homolog Mi-copy1, *S. * *lycopersicum*	5	9
cTOF26E9	C144413	Prf, *S. pimpinellifolium*	5	2
cTOE1K1	C136851	Spermidine synthase, *S. lycopersicum*	5	4
cTOE7J7b	C139397	Endo-1,4-beta-glucanase, *S. lycopersicum*	5	6
cTOF29B13	C145236	Metallothionein-like protein type 2 a, *S. lycopersicum*	5	2
cTOF33C3	C146601	Serine/threonine protein kinase Pto, *S. lycopersicum*	5	10
cTOF23J19	C143585	Heat shock protein 90, *S. lycopersicum*	5	4
cLEG32E10	C34795	Lipoxygenase B, *S. lycopersicum*	6	6
cTOF8F19	C148467	Ascorbate peroxidase, *S. lycopersicum*	6	2
cLEZ16H16	C99197	Contains similarity to disease resistance response protein, *Pisum sativum*	6	1
cLED11A2	C15134	Mitogen-activated protein (MAP) kinase 3, *C. annuum*	6	2
cLEW22D11a	C90352	4-coumarate:coenzyme A ligase, *N. Tabacum*	6	10
cLEY21L21	C97473	Disease resistance gene homolog Mi-copy1, *S. lycopersicum*	6	6
cLEW22N22	C90504	Ethylene-responsive element binding factor 6-N. sylvestris	6	3
cTOF34C13	C146804	Peroxiredoxin Q-like protein, *A. thaliana*	7	1
cLEN14F9	C66474	Sucrose-phosphate synthase, *S. lycopersicum*	7	1
cTOF21F12	C142982	Dehydroquinate dehydratase/shikimate, NADP oxidoreductase, *S. lycopersicum*	7	9
cLEN13G22	C66246	1-aminocyclopropane-1-carboxylate oxidase, *S. lycopersicum*	7	4
cLEY22L20	C97674	Peroxidase precursor, *S. lycopersicum*	7	3
cTOE15M9	C136013	MYB-related transcription factor VlMYBB1-1, *Vitis labrusca* × *V. vinifera*	7	6
cLEG34O20	C35423	UDP-glucose:salicylic acid glucosyltransferase, *N. tabacum*	7	4
cLEN14C8	C66419	PR-related protein, PR P23 (salt-induced protein), *S. lycopersicum*	8	3
cTOF9D16	C148734	Pathogenesis-related protein 5-1, *S. lycopersicum*	8	1
cTOF28D12	C144993	Polyphenol oxidase E, chloroplast precursor, *S. lycopersicum*	8	**7**
cLEN10H3	C65539	Heat shock factor protein HSF8 (Heat shock transcription factor 8), *S. lycopersicum*	8	2
cLEI16E21	C47449	Cold-induced glucosyl transferase, *S. lycopersicum*	8	3
cTOF2N15	C145786	Osmotin-like protein OSML13 precursor (PA13), *S. lycopersicum*	8	3
cTOE23J12	C137767	Monodehydroascorbate reductase, *S. lycopersicum*	8	3
cLED27C20	C19537	DNADPH oxidase; gp91-phox homolog, *S. lycopersicum*	8	1
cLER14J12	C70373	WRKY transcription factor IId-1 splice variant 2, *S. lycopersicum*	8	1
cTOF2L16	C145747	Phenylalanine ammonia-lyase (PAL), *S. lycopersicum*	8	1
cTOD3N7	C132799	Endo-1,4-beta-glucanase, *S. lycopersicum*	8	2
cLEX11E19	C92435	Putative NADH-ubiquinone oxireductase, *A. thaliana*	9	1
cTOE10J18	C134749	PR protein sth-2, *S. Tuberosum*	9	3
cLEC13E21	C1592	P14 (PR-Protein), *S. lycopersicum*	9	3
cLEC6M14	C10964	PR-protein sth-2, *S. Tuberosum*	9	5
cLER14J6	C70387	Hexose transporter, *S. lycopersicum*	9	1
cTOF19O3	C142383	Hydroxyproline-rich glycoprotein homolog, *A. thaliana*	9	3
cLEZ6E21b	C100278	Ubiquitin, *S. lycopersicum*	10	4/5
cLED18G6	C17041	Similar to WRKY-like drought-induced protein, *Retama raetam*	10	6
cTOD4I20	C133021	Tyrosine aminotransferase, *A. thaliana*	10	2
cLHT11J12	C100975	Diacylglycerol kinase, *S. lycopersicum*	10	2
cLER4F5	C73337	Ferredoxin-I chloroplast precursor *S. lycopersicum*	10	4
cTOF30K21	C146034	Chloroplast ferredoxin I, *S. lycopersicum*	10	>10
cTOF22M16	C143336	NADH-ubiquinone oxidoreductase 23 kDa subunit, *S. lycopersicum*	10	1
cLEN14K6	C66563	Multiresistance protein homolog, *A. thaliana*	10	3/2
cLEX10N16	C92314	PR protein, *S. lycopersicum*	10	>10
cLEC18O1	C3034	Basic 30kDa endochitinase precursor, *S. lycopersicum*	10	>10
cTOF31H10	C146231	Catechol O-methyltransferase, *N. tabacum*	10	8
cLEN9P2	C69374	Multiresistance protein homolog, *A. thaliana*	10	2
cLED13I7	C15652	Resistance complex protein I2C-1, *S. lycopersicum*	11	7
cTOF28I23	C145097	Resistance complex protein I2C-5, *S. pimpinellifolium*	11	>10
cLEZ6E21a	C100278	Ubiquitin, *S. lycopersicum*	11	4/5
cTOF29F6b	C145330	10-hydroxygeraniol oxidoreductase, -*S. lycopersicum*	11	7
cLEC14I18a	C1998	Resistance complex protein I2C-2, *S. lycopersicum*	11	>10
cLEC14I18b	C1998	Resistance complex protein I2C-2, *S. lycopersicum*	11	>10
cLEM22K17	C62708	9-cis-epoxycarotenoid dioxygenase, *S. lycopersicum*	11	7
cLED23K21	C18512	Resistance complex protein I2C-5, *S. lycopersicum*	11	>10
cLES18N16	C76694	Phosphatidylinositol 4-kinase, *S. tuberosum*	11	2
cTOS21D14	C163579	WRKY transcription factor IId-2, *S. lycopersicum*	12	1
cLPT1G11	C109877	S-adenosyl-l-homocysteine hydrolase, *S. lycopersicum*	12	4
cLEZ15E8	C98979	Extensin class I, *S. Lycopersicum*	12	>10
cLEW25D9	C90989	Glutamine synthetase, *S. lycopersicum*	12	3

^a^Solanaceae Genome Network (SGN) can be accessed at http://www.sgn.cornell.edu/. ^b^The putative function of each EST has been derived from Computational Biology and Functional Genomics Laboratory web site (http://compbio.dfci.harvard.edu/tgi/cgi-bin/tgi/est_report.pl), used to be maintained at The Institute for Genomic Research (TIGR). (Computational Biology and Functional Genomics Laboratory). ^c^The exact or approximate copy number of ESTs in tomato genome was determined based on the number of hybridized bands on Southern blot gels and may be varied in different labs. Where there is a “/” sign, the figures in the left side denote the number of copies in *S. lycopersicum* parent and those in the right side denote the number of copies in *S. pimpinellifolium* parent.

**Table 2:** Oligonucleotide primers designed based on the conserved amino acid sequences within the *LRR*, *NBS,* and *Pto* protein domains encoded by various *R*-genes.

Group	Primers	Sequences (5′-3′)^a^	Design basis	References
LRR	CLRR for	TTTTCGTGTTCAACGACG	LRR domain of the tomato *Cf*-*9* gene conferring resistance to *Cladosporium fulvum*	[30]
	CLRR rev	TAACGTCTATCGACTTCT		
		
	NLRR for	TAGGGCCTCTTGCATCGT	LRR domain of the tobacco *N* gene conferring resistance to TMV	
	NLRR rev	TATAAAAAGTGCCGGACT		
		
	RLRR for	CGCAACCACTAGAGTAAC	LRR domain in the *RPS2* gene conferring resistance to *Pseudomonas syringae* in *Arabidopsis*	
	RLRR rev	ACACTGGTCCATGAGGTT		
		
	XLRR for	CCGTTGGACAGGAAGGAG	LRR domain of the rice *Xa21* gene conferring resistance to *Xanthomonas campestris* pv *oryzae*	
	XLRR rev	CCCATAGACCGGACTGTT		
				

NBS	ANo. 2	TATAGCGGCCGCIARIGCIARIGGIARNCC	Conserved P-loop and hydrophobic NBS regions of the *N* and *RPS2* genes from tobacco and *Arabidopsis* respectively	[29]
	ANo. 3	ATATGCGGCCGCGGIGGIGTIGGIAARACNAC		
			
	S1	GGTGGGGTTGGGAAGACAACG	Hydrophobic domain and P-loop of conserved NBSs from the *N* and *RPS2* genes from *Arabidopsis* and the *L6* gene from flax conferring resistance to rust	[18, 31]
	S2	GGIGGIGTIGGIAAIACIAC		
	AS1	CAACGCTAGTGGCAATCC		
	AS3	IAGIGCIAGIGGIAGICC		

PtoKin	Ptokin1	GCATTGGAACAAGGTGAA	Serine/threonine protein kinase domain of the *Pto* gene conferring resistance to the bacterial pathogen *Pseudomonas syringae* pv *tomato* in tomato	[30]
	Ptokin2	AGGGGGACCACCACGTAG		
	Ptokin3	TAGTTCGGACGTTTACAT		
	Ptokin4	AGTGTCTTGTAGGGTATC		
			
	RLK for	GAYGTNAARCCIGARAA	Serine/threonine kinase sequence subdomains of the wheat *Lr10* gene conferring resistance to *Puccinia recondita*	[20]
	RLK rev	TCYGGYGCRATRTANCCNGGITGICC		
				

^a^Code for mixed bases: D = A/G/T; I = Inosine; N = A/G/C/T; R = A/G; Y = C/T.

**Table 3:** Significant deviations from the expected 3 : 1 and 1 : 1 ratios in the *Solanum lycopersicum* × *S*. *pimpinellifolium* F_2_ population (L: *lycopersicum* allele, PM: *pimpinellifolium* allele).

Locus	Chromosome	Genotype					
		L/L	L/PM	PM/PM	PM/−	L/−	*χ* ^2^*
AN23_240	1	16	0	0	141	0	18.36
S13_310	1	16	0	0	139	0	17.81
S11_180	1	16	0	0	140	0	18.09
S11_150	1	17	0	0	139	0	16.55
S11_200	1	17	0	0	139	0	16.55
NBS4_300	1	18	78	40	0	0	10.06
TG125	1	18	84	43	0	0	12.27
cTOF3A14	1	20	91	43	0	0	11.96
TG132	3	28	85	57	0	0	9.89
TG66	3	22	90	44	0	0	9.90
CT225B	3	15	91	34	0	0	17.76
cLEX10F20	3	24	97	36	0	0	10.55
CT82	3	24	98	38	0	0	10.55
cLER17H16	3	56	69	32	0	0	9.64
cLEW24M21	4	36	63	55	0	0	9.78
CT178	4	35	74	60	0	0	10.01
C25	4	35	67	56	0	0	9.23
CT73	4	42	67	58	0	0	9.59
CT93	5	51	98	17	0	0	19.35
cLER5E19	5	0	0	16	0	138	17.53
TG503	5	50	87	17	0	0	16.74
cTOF33C3	5	45	86	14	0	0	18.28
cTOF26E9	5	51	100	16	0	0	21.19
TG96	5	50	79	14	0	0	19.70
cTOF23J19	5	35	82	13	0	0	16.34
XLRR380	5	0	0	14	0	143	21.66
TG351	5	44	87	18	0	0	13.27
cTOC2J14a	5	34	99	12	0	0	26.05
cTOC2J14b	5	59	79	13	0	0	28.35
cTOF29B13	5	59	78	14	0	0	26.99
TG185	5	46	75	12	0	0	19.56
CT285	6	61	72	27	0	0	16.05
TG356	6	82	53	16	0	0	71.11
cLEW22D11a	6	92	44	13	0	0	108.74
cLEW22N22	6	103	39	6	0	0	160.26
TG365	6	118	41	7	0	0	190.95
TG253	6	132	30	4	0	0	265.08
C54	6	154	11	2	0	0	402.59
TG279	6	156	4	1	0	0	443.84
cLEZ16H16	6	142	22	1	0	0	329.72
TG477	6	135	24	1	0	0	302.85

*All *χ* ^2^ values significant at *P* < .01.

**Table 4:** Comparison of map distances based on common marker intervals between three molecular linkage maps of tomato^a^.

Interval	Chr.	Marker interval map distance (cM)				
		(L × PM3)^b^	(L × PM2)^c^	(L × PM3)/(L × PM2)	(L × P)^d^	(L × PM3)/(L × P)
TG70-TG273	1	24.3	23.8	1.0	8.9	2.7*
TG554-TG453	2	6.6	—	—	0.0	NA*
TG453-TG145	2	0.4	—	—	6.8	0.1*
TG145-CT103	2	10.3	—	—	10.1	1.0
CT176-TG582	2	5.6	18.9	0.3*	15.5	0.4*
CT59-TG620	2	2.6	7.0	0.4*	0.0	NA*
TG114-TG132	3	0.0	8.9	N/A*	15.4	NA*
TG66-CT225B	3	6.6	—	—	1.8	3.7*
CT225B-CT82	3	9.8	—	—	6.3	1.6
CT82-TG515	3	12.7	14.8	0.9	3.9	3.3*
TG123-TG182	4	12.5	—	—	14.2	0.9
TG182-TG609	4	6.7	—	—	5.5	1.2
TG609-CT178	4	11.8	—	—	11.1	1.1
CT167-CT93	5	19.3	8.9	2.2*	12.9	1.5
TG503-TG96	5	2.5	—	—	3.2	0.8
TG274-TG590	6	4.6	—	—	10.4	0.4*
TG356-TG365	6	13.0	11.9	1.1	4.1	3.2*
C54-TG279	6	4.0	10.2	0.4*	—	NA
TG183-TG128	7	15.7	—	—	2.3	6.8*
TG128-CT226	7	3.1	3.5	0.9	1.6	1.9
TG128-TG174	7	19.3	13.6	1.4	10.7	1.8
TG176-CD40	8	8.7	—	—	0.0	NA*
CT265-TG294	8	12.8	9.6	1.3	13.1	1.0
TG486-CD3	9	1.7	—	—	1.3	1.3
CD3-CT279	9	11.3	—	—	5.6	2.0*
CT279-TG35	9	1.9	—	—	0.0	NA
TG408-CD34	10	4.9	—	—	22.9	0.2*
CD34-TG403	10	30.1	37.5	0.8	7.4	4.1*
TG629-TG497	11	0.0	—	—	0.0	NA
TG30-CT65	11	16.7	—	—	3.9	4.3*
TG68-CT79	12	3.3	6.7	0.5*	14.4	0.2*
CT99-TG618	12	5.4	—	—	0.8	6.8*
TG618-TG111	12	12.5	—	—	6.1	2.0*
TG111-TG565	12	3.0	—	—	0.0	NA*
CT156-TG473	12	6.1	—	—	19.1	0.3*
TG473-CD2	12	0.0	—	—	1.8	0.0

^a^Only common marker intervals that were different in length by at least twofold between L × PM1 and either L × PM2 or L × P linkage maps are shown. ^b^L × PM3: *Solanum lycopersicum* (NCEBR-1) × *S. pimpinellifolium* (LA2093) map (present map). ^c^L × PM2: *S. lycopersicum* (NC84173) × *S. pimpinellifolium* (LA722) map [7]. ^d^L × P: *S.* *lycopersicum* (VF36-*Tm*2) × *S. pennellii* (LA716) map [5]. *Difference in interval length by at least twofold. Dashes (—) indicate no common interval for comparison. NA indicates a number divided by 0.0, 0.0 over a number, or no comparison was made.

**Table 5:** Pairwise comparison of the present map (L × PM3) with other maps of tomato for individual chromosome lengths based on orthologous markers.

					Chromosome length (cM)									
Linkage map^a^	1	2	3	4	5	6	7	8	9	10	11	12	Average	Total
L × PM3	102.9	92.6	85.3	72.2	70.6	74.6	69.8	86.6	96.1	80.6	88.3	83.4	83.6	1003.0
L × PM2	129.7	121.9	133.8	108	94.1	82.8	91.3	64.4	104.8	84.9	78.2	92.6	98.9	1186.5
L × PM1	149.6	98.2	116.6	97.2	108.2	85.2	116.4	86.1	104.2	101.5	107	105.2	106.3	1275.4
L × P	133.5	124.2	126.1	124.8	97.4	101.9	91.6	94.9	111	90.1	88	93.1	106.4	1276.6
L × PM3/L × PM2	0.8	0.8	0.6	0.7	0.8	0.9	0.8	1.3	0.9	0.9	1.1	0.9	0.8	
L × PM3/L × PM1	0.7	0.9	0.7	0.7	0.7	0.9	0.6	1.0	0.9	0.8	0.8	0.8	0.8	
L × PM3/L × P	0.8	0.7	0.7	0.6	0.7	0.7	0.8	0.9	0.9	0.9	1.0	0.9	0.8	

*L × PM3, *S. lycopersicum* (NCEBR-1) × *S. pimpinellifolium* (PSLP125) map (the present map); L × PM2, *S. lycopersicum* (NC84173) × *S. pimpinellifolium* (LA722) map [7]; L × PM1, *S. lycopersicum* (M82-1-7) × *S. pimpinellifolium* (LA1589) map [6]; E × P, *S. lycopersicum* (VF36-Tm2) × *S. pennellii* (LA716) map [25].
